# Identification of Immune-Related Subtypes and Characterization of Tumor Microenvironment Infiltration in Kidney Renal Clear Cell Carcinoma

**DOI:** 10.3389/fgene.2022.906113

**Published:** 2022-06-29

**Authors:** Huisheng Qin, Tiancheng Wang, Hui Zhang

**Affiliations:** ^1^ Department of Urology, Affiliated Hospital of Jining Medical University, Jining, China; ^2^ Department of General Surgery, The First Affiliated Hospital of Shandong First Medical University & Shandong Provincial Qianfoshan Hospital, Jinan, China; ^3^ Department of Oncology, Affiliated Hospital of Jining Medical University, Jining, China

**Keywords:** tumor microenvironment, immune score, TCGA, kidney renal clear cell carcinoma, TME

## Abstract

**Background:** Tumor microenvironment (TME) plays indisputable role in the progression of cancers. Immune cell infiltration (ICI) in TME was related to the prognosis of tumor patients. In this paper, we identified the pattern of immune-related ICI subtypes based on the TME immune infiltration pattern.

**Methods:** The data from kidney renal clear cell carcinoma data (KIRC) was downloaded from the TCGA database. The distinct ICI subtypes were identified using CIBERSORT and ESTIMATE algorithms. The gene subgroups were identified based on DEGs in ICI subtypes. The single sample gene set enrichment analysis (ssGSEA) was used to ascertain the ICI score. Kaplan-Meier curve with log-rank test was conducted to analyze the survival probability of patients with KIRC in different subtypes.

**Results:** The patients with high ICI scores exhibited a longer survival time and lower expression of checkpoint-related and immune activity-related genes. The high ICI score clusters were positively related to TMB. The patients in the low TMB subgroups have a favorable prognosis. The prediction ICI score did not affect the TMB status, and the low TMB subgroups + low/high ICI score subgroups exhibited better survival.

**Conclusion:** In all, the tumor immune microenvironment, ICI score, and TMB were important determinants of KIRC patients’ survival outcomes. The TMB + ICI score may be a potential biomarker for predicting the prognosis of patients and for targeted immunotherapies to treating KIRC.

## Introduction

Kidney renal clear cell carcinoma (KIRC) is one of the most common malignant tumors. Among the subtypes of kidney cancer, renal cell carcinoma (RCC) is the main subtype, which is a malignant tumor originating from the renal tubular epithelium, accounting for about 85% of renal cancers (Moch et al., 2016). Ultimately, approximately 40% of patients die from the tumor progression of renal cancer, making renal cancer the main cause of death among urologic tumors (Ramana, 2012). In recent years, the age of onset of renal cancer has tended to be younger. Renal cancer usually has insidious symptoms in the early stage and is difficult for early diagnose. Even with the rapid development of modern imaging technology, 20%–30% of patients still have local or distant metastasis at the initial diagnosis (Guo et al., 2021). Even after surgical treatment, recurrence or metastasis still occurs in about 20% of patients (Jing et al., 2021). Metastatic renal cancer is not sensitive to radiotherapy and chemotherapy, and the effective rate of immunotherapy with interferon and interleukin-2 (IL-2) as first-time clinical treatment is only 15% (Zou et al., 2019). Although the use of tyrosine kinase inhibitors has improved the prognosis of advanced/metastatic RCC in recent years, the prognosis is still poor (Nasso et al., 2021). Therefore, there is an urgent need for exploring more effective therapeutic targets for treatments of advanced/metastatic renal cancer.

Renal cancer is considered to be an immunogenic tumor, characterized by many inflammatory cells such as T cells, NK cells, dendritic cells (DC), and macrophage around renal cancer, which play an effective anti-tumor effect mainly through the variation of DC and the inability of CD8^+^ T cells to evade immune surveillance and interfere with the immune system [9]. Various immunotherapy methods are emerging to further understand kidney cancer and the immune system. In 2005, for example, IL-2 and IFN-α were used as standard treatments for metastatic kidney cancer (de Velasco et al., 2015). Importantly, in recent years, the discovery of immune checkpoint inhibitors (such as anti-PD-1 antibodies, and anti-PD-L1 antibodies) in immune pathways has led to breakthroughs in the treatment of advanced/metastatic renal cancer (Zhang et al., 2016). Combination immunotherapy is recommended as the first-line treatment for advanced renal cell carcinoma. However, due to the rich blood supply for advanced renal cell carcinoma, anti-angiogenesis is still an important treatment option. Therefore, how to screen the treatment subtype population has become a very important clinical issue.

The tumor microenvironment (TME) is the internal environment in which tumor cells occur and survive and are closely related to tumor cells (Mu et al., 2021). TME also includes the cellular matrix, microvessels, and various biomolecules infiltrated in the surrounding area, which is collectively referred to as the extracellular matrix (ECM) (Tan et al., 2020). Tumor cells acquire cell-autonomous proliferation and viability by activating oncogenes and tumor suppressor genes in TME (Hitosugi and Chen, 2014). Emerging evidence revealed that the TME can create survival conditions for cancer stem cells or initially transformed cells and can facilitate tumor cell metastasis (Zeng et al., 2020). As two important components of the ECM in the TME, immune cells and stromal cells have extensive interactions with tumor cells, including early tumor recruitment and activation of stromal cells to form primitive precancerous cells (Guarino et al., 2019). Therefore, analysis of immune- and stomal-related gene expression profiles is crucial to improve the ability to guide and predict immune response and prognosis for patients with renal cancer.

The acquisition of the phenotype of the high metastatic ability of tumor cells is based on the abnormal changes of a series of metastasis-related genes. Herein, we identified four distant immune cell infiltration (ICI) groups using the CIBERSORT algorithm. The stromal score and immune score were calculated using the ESTIMATE algorithm of 530 KIRC tumor specimens. The patients’ survival status was evaluated in three gene clusters. The ICI score was established to characterize the immune landscape of KIRC, which may be used as an effective prognostic predictor for immunotherapy and novel directions for immunotherapy strategies for KIRC patients.

## Materials and Methods

### Study Data and Pre-Processing

KIRC data, including transcriptome data and clinical data, were acquired from the publicly available dataset TCGA. The RNA-Seq data of the TCGA-KIRC cohort were obtained using the R package “TCGAbiolinks.” The TPM values were transformed from the FPKM values. Samples with incomplete clinical and survival information were removed. Finally, a total of 530 KIRC samples were obtained for subsequent analysis. The mutation data were obtained from cBioPortal.

### Unsupervised Clustering Analysis

The CIBERSORT algorithm was performed to quantify the ICI score in renal cell carcinoma (Newman et al., 2015). The ESTIMATE algorithm was run to calculate the stromal score and immune score of the samples (Yoshihara et al., 2013). Unsupervised clustering was performed to group patients into distinct ICI subtypes. The number of clusters and stability were determined with the help of consensus clustering using the R package (Wilkerson and Hayes, 2010). The optimal cluster number was determined by the clustering score for the cumulative distribution function (CDF) curve and the relative changes in the area under the CDF curve.

### Identification of DEGs

The patients with RCC were grouped into four distinct ICI subtypes to recognize ICI-related genes in RCC. To explore DEGs among different ICI subtypes, the R package “limma” was performed. Significance criteria for determining DEGs were adjusted *p* value less than 0.05 with a fold change of more than 4.

### Immune Cell Infiltration Score Generation

According to the unsupervised clustering of DEGs, the ICI score was calculated to quantify ICI patterns of RCC. The RCC samples were redistributed into different gene clusters. The Pearson correlation analysis on DEGs and clustering features was performed and the gene expression values positively correlated with clusters were called the ICI genotype A and the remaining DEGs were called ICI genotype B, respectively. At the same time, to reduce the redundant genes, the Boruta algorithm was used to reduce the dimensionality of ICI gene validation A and B. The signature scores were calculated using ssGESA using the R package “GSVA.”

### Somatic Gene Mutation Analysis

The mutation data of TCGA-KIRC was obtained from cBioPortal. The patients were classified into low and high ICI score subgroups to determine the relationship between somatic gene mutation and ICI score. Then the R package “maftools” was used to identify driver genes in the low and high ICI subgroups. The top 20 driver genes with the higher mutation frequency were further analyzed.

### Collection of Clinical and Genomic Data With Immunotherapy

The prognostic prediction significance of the ICI score was validated. The R package “IMvigor210CoreBiologies” was used to acquire the expression data and clinical annotations of the immunotherapy cohort. The gene expression data were transformed into TPM values for subsequent analysis.

### Statistical Analysis

R version 4.0.2 was conducted for all statistical analyses. The group comparisons were carried out using the Wilcoxon test or the Kruskal-Wallis test. Kaplan-Meier curve was conducted to evaluate survival difference with the log-rank test in patients with RCC. Pearson correlation analysis was used to analyze the correlation coefficient. A *p*-value less than 0.05 indicates statistical significance.

## Results

### Data Source and Immune Cell Infiltration Subtypes in Kidney Renal Clear Cell Carcinoma

**FIGURE 1:**
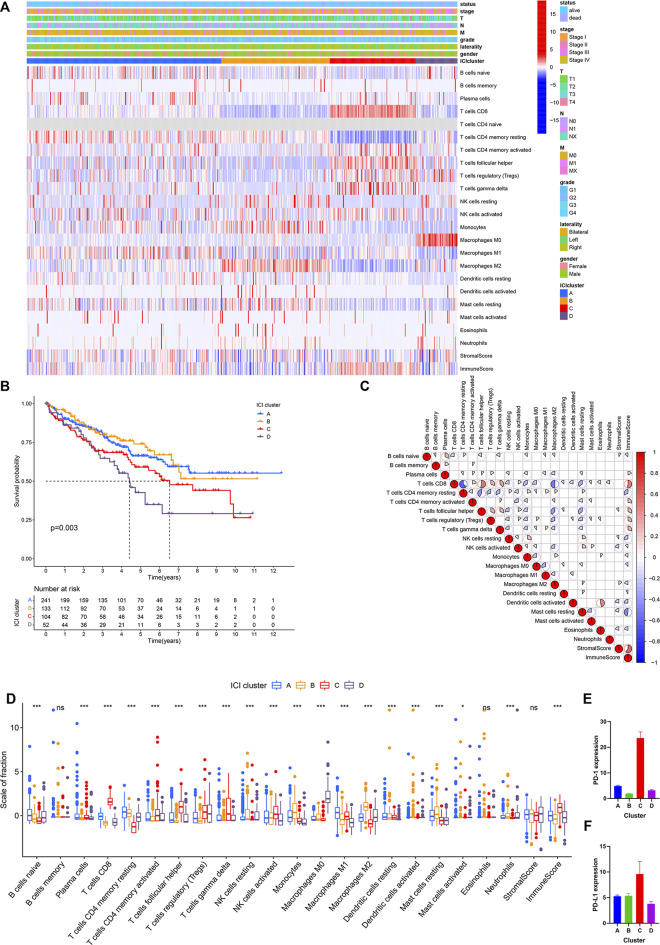
(Continued.)The ICI score subtypes in KIRC. **(A)** The KIRC data were downloaded from the TCGA database and grouped into four ICI subtypes. **(B)** Kaplan-Meier curve was conducted to analyze the survival probability in four ICI clusters (log-rank test *p* = 0.003). **(C)** Immune cellular interaction of the ICI score subtypes. **(D)** The difference analysis of the fraction of immune cells, immune score, and stromal score in four ICI clusters. *p < 0.05, ***p < 0.001. **(E,F)** The difference in PD-L1 **(E)** and PD1 **(F)** expression among four ICI clusters.,

Publicly available data for the 530 cases of KIRC, including RNA-seq and clinical data were obtained from the TCGA database. The CIBERSORT algorithm was used to estimate the abundance of 22 immune cells. The ESTIMATE algorithm was used to establish the enrichment scores of stromal cells and immune cells. There are 18 signatures for clustering analysis. The results were shown in Supplementary Table S1. Based on the 18 signatures in 530 KIRC patients, four distinct ICI subtypes were identified (Figure 1A; Supplementary Figure S1). The overall survival of the four ICI subtypes was analyzed (log-rank test, *p* = 0.003, Figure 1B). The correlation among 22 immune cells was analyzed and the immune cell interaction in the TME was visualized in Figure 1C. Subsequently, the TME of the four distinct ICI subtypes was compared to elucidate the difference in all the ICI subtypes. The infiltration levels of macrophages M1, plasma cells, dendritic cells activated, and activated mast cells were higher in ICI cluster A. ICI cluster B was marked by increased resting NK cells, monocytes, macrophages M2, and resting mast cells. Patients in ICI cluster C were characterized by high infiltration level of CD8^+^ T cells, activated CD4 memory T cells, *γδ* T cells, activated NK cells, and immune score. ICI cluster D was marked with high macrophage M0 and had the shortest overall survival time (Figure 1D). Then, PD1 and PD-L1 levels were analyzed and compared among the ICI subtypes. As indicated in Figures 1E,F, the ICI subtype C exhibited the highest expression levels of PD1 and PD-L1.

### Identification of Gene Subtypes

**FIGURE 2:**
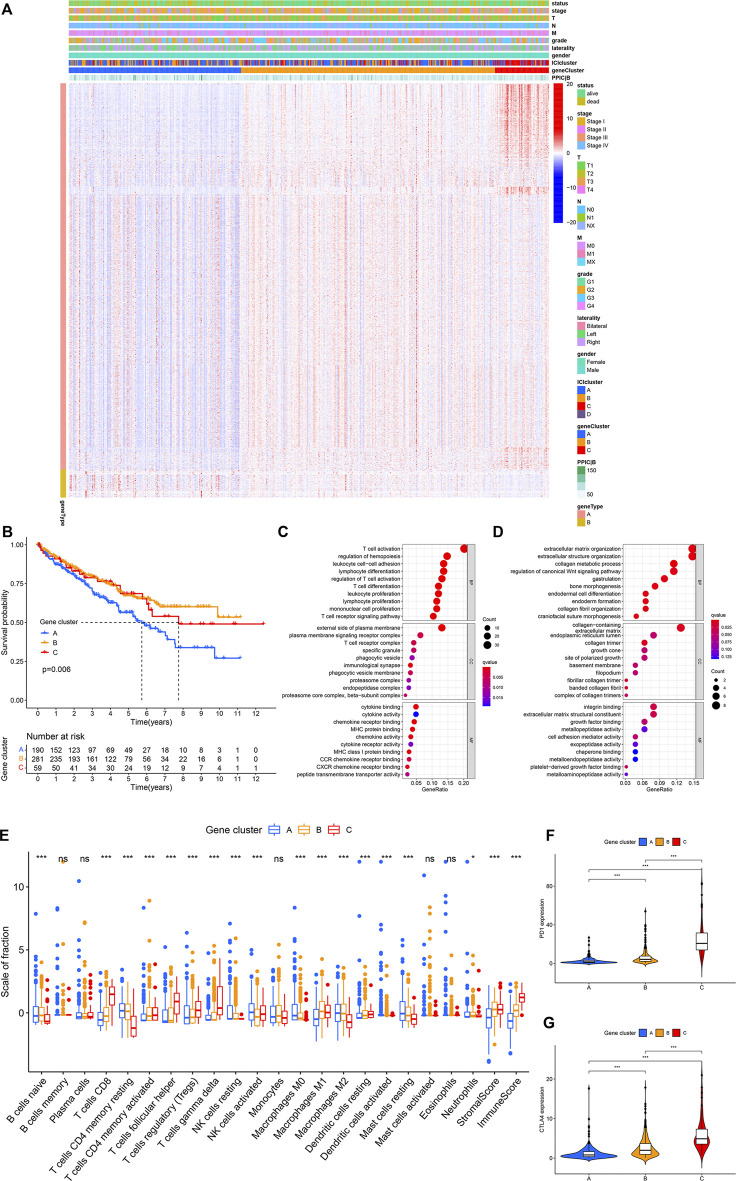
(Continued.)The gene subtypes were identified based on DEGs in the TCGA-KIRC cohort. **(A)** Consensus clustering of DEGs among four ICI subtypes. **(B)** Kaplan-Meier analysis was conducted to analyze the survival probability of the three gene clusters (log-rank test *p* = 0.006). **(C,D)** GO enrichment analysis of signature genes. **(E)** The infiltration of immune cells among three gene clusters. *p < 0.05, ***p < 0.001. **(F,G)** The difference in the expression of PD1 **(F)** and CTLA4 **(G)** among three gene clusters. ***p < 0.001.,

Subsequently, the R package “limma” was used to determine transcriptome differences among ICI subtypes (Ritchie et al., 2015). As listed in Supplementary Table S2, 1,398 DEGs were identified (*p* < 0.001, |logFC| > 4). The unsupervised clustering was applied to group patients into three subtypes based on 1,398 DEGs (Supplementary Figure S2), and gene clusters A–C (Figure 2A). Gene cluster A had a lower expression of gene signature A and gene signature B. Gene cluster B exhibited a higher expression level of gene signature A and B. Gene cluster C had a higher expression of gene signature A and a lower expression level of gene signature B (Figure 2A). The patients with gene cluster A had the worse prognosis (log-rank test, *p* = 0.006, Figure 2B). The gene ontology (GO) enrichment analysis results showed the enriched biological processes in gene signatures, which were summarized in Figures 2C,D, respectively. The gene A cluster A had high resting NK cells, macrophages M0, and resting mast cells. Gene B cluster exhibited a higher macrophages M1 and stromal score. The gene C cluster displayed a higher immune score, *γδ* T cells, T cells follicular helper, and CD8^+^ T cells (Figure 2E). Additionally, gene cluster C had the highest expression levels of immune checkpoints PD1 and CTLA4 (Figures 2F,G).

### Establishment of the Immune Cell Infiltration Scores

**FIGURE 3:**
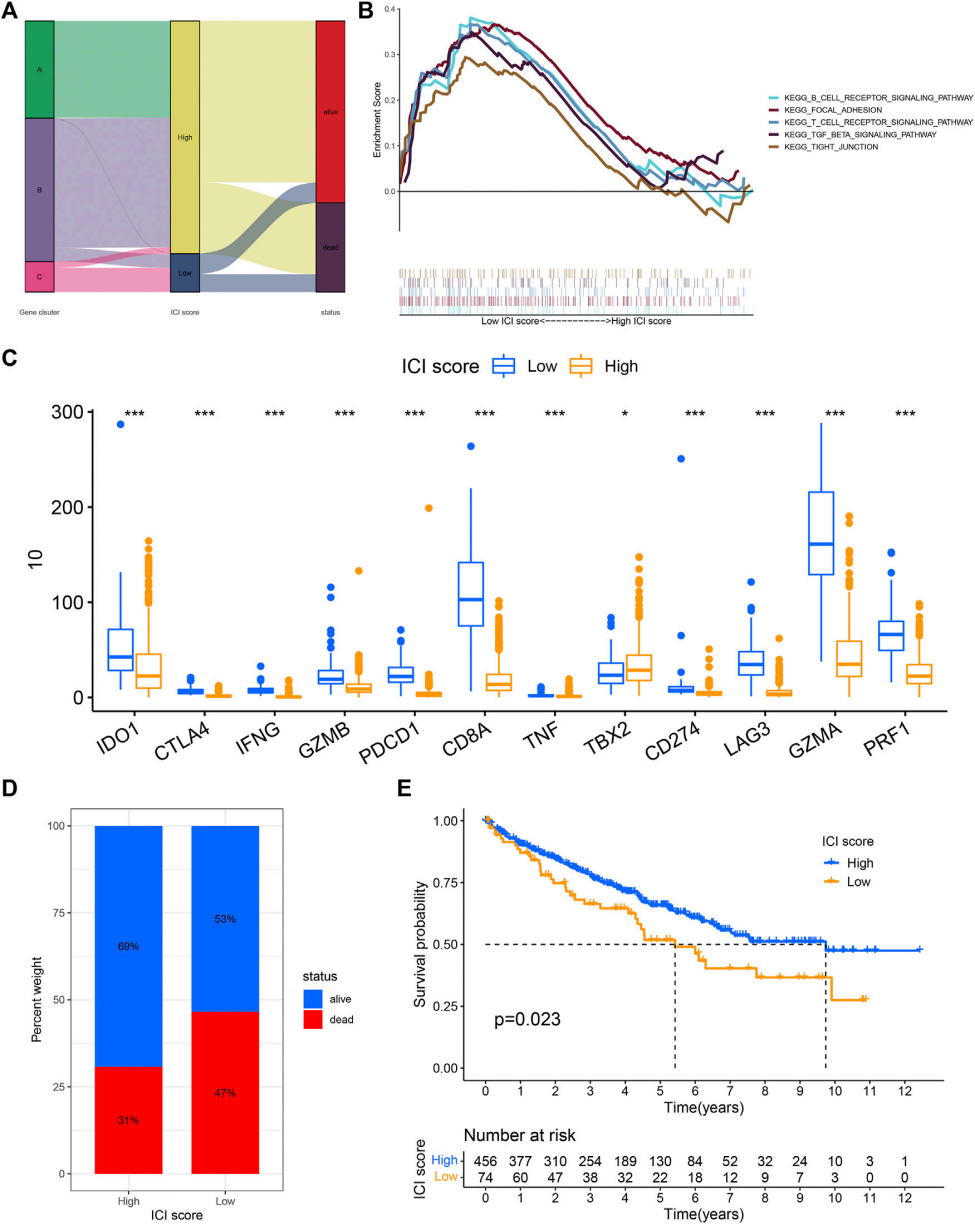
ICI score was constructed. **(A)** Sankey diagram showed the relationship between gene cluster, ICI score, and survival status. **(B)** The gene set enrichment analysis (GSEA) in the low and high ICI score subgroups. **(C)** The difference in the expression of immune activity-related genes and immune checkpoint-related genes between low and high ICI score subgroups. **(D,E)** Kaplan-Meier curve analysis for low and high ICI score subgroups in the KIRC cohort (log-rank test *p* = 0.023).

The PCA analysis was used to calculate the score of gene signatures A and B, and the ICI score was obtained after subtraction score B from score A. The ICI score was listed in Supplementary Table S3. The Boruta R package was used to search for characteristic genes in both A and B groups and the genes were listed in Supplementary Table S4. KIRC patients were classified into low and high ICI score subgroups. The patients’ distribution in three gene subtypes and two score subgroups is shown in Figure 3A. Gene set enrichment analysis (GSEA) and KEGG were executed and the results were displayed in Figure 3B. The immune checkpoint- and immune activity-related genes were compared between low and high ICI score groups. The data revealed significant downregulation of most genes in the high ICI score group, while only TBX2 expression was increased (Figure 3C). The survival curve demonstrated that the survival probability of patients in the low ICI score group was poor compared with that in the high ICI score group (log-rank test, *p* = 0.023; Figures 3D,E).

### The Relationship Between Immune Cell Infiltration Score and Clinical Characteristics of Patients and Somatic Mutation

**FIGURE 4:**
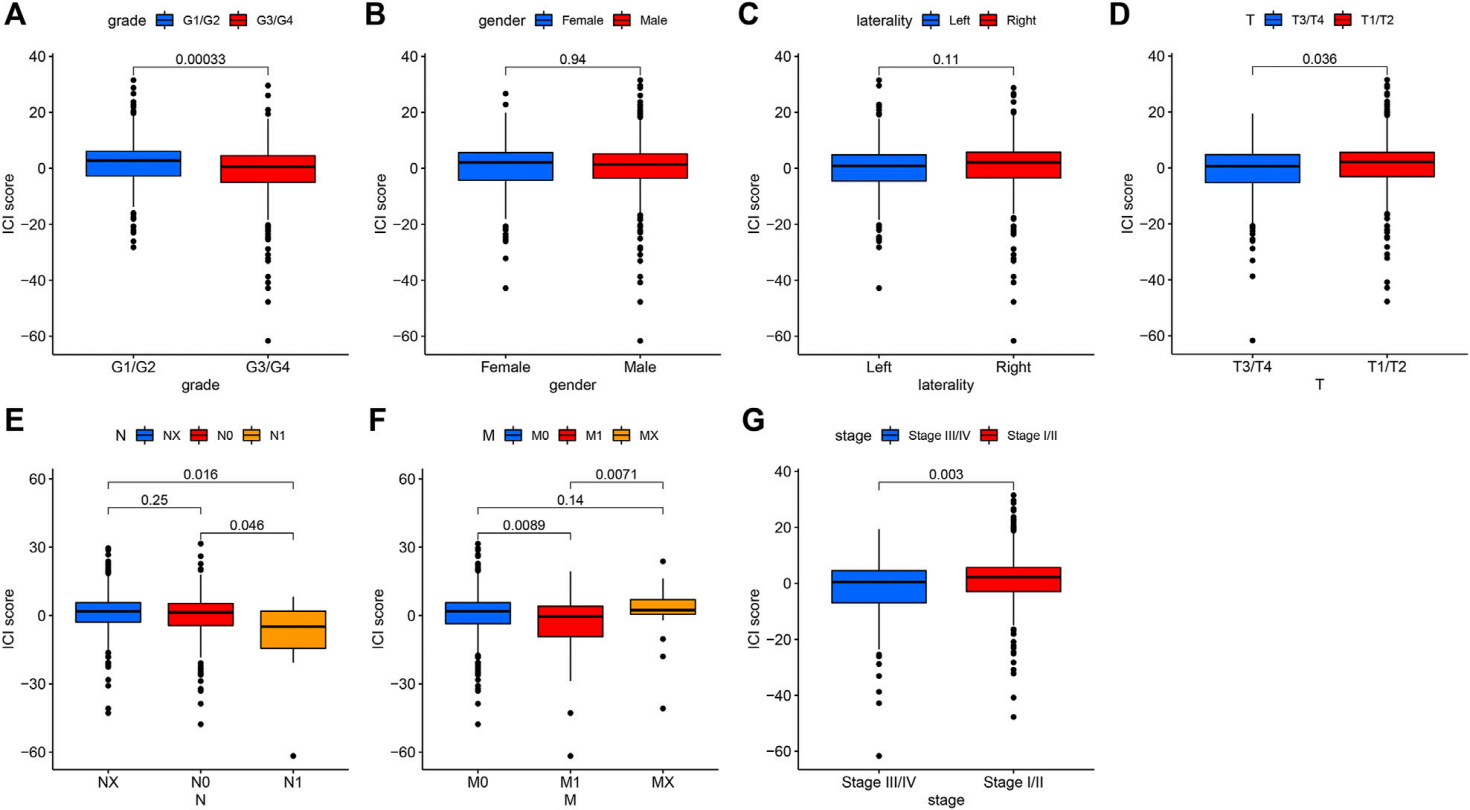
The association between ICI scores and some clinical characteristics of patients with KIRC. **(A)**. The relationship between ICI score and Grade. *p* = 0.00033. **(B)** Gender parameter has no significant relationship with ICI score. **(C)** The association between laterality and ICI scores. **(D)** The patients with T3/T4 had low scores. *p* = 0.036. **(E)** The positive node metastasis (N1) was significantly related to low ICI scores. *p* = 0.016. **(F)** The relationship between metastasis (M) with ICI scores. *p* = 0.0089. **(G)** The low ICI score was observed in stages III/IV. *p* = 0.003.

We further analyzed the association between ICI score and clinical parameters [such as grade, gender, laterality, distant metastasis (M), and node metastasis (N)] of the patients with KIRC. The results revealed that patients with grade G3/G4 (Figure 4A), T3/T4 (Figure 4D), node metastasis (Figure 4E), distant metastasis (Figure 4F), and stage III/IV (Figure 4G) had a low ICI score (*p* < 0.05), while no association was observed between ICI score with gender (Figure 4B) and laterality (Figure 4C), which suggest that the ICI score has a relationship with the progression of KIRC.

**FIGURE 5:**
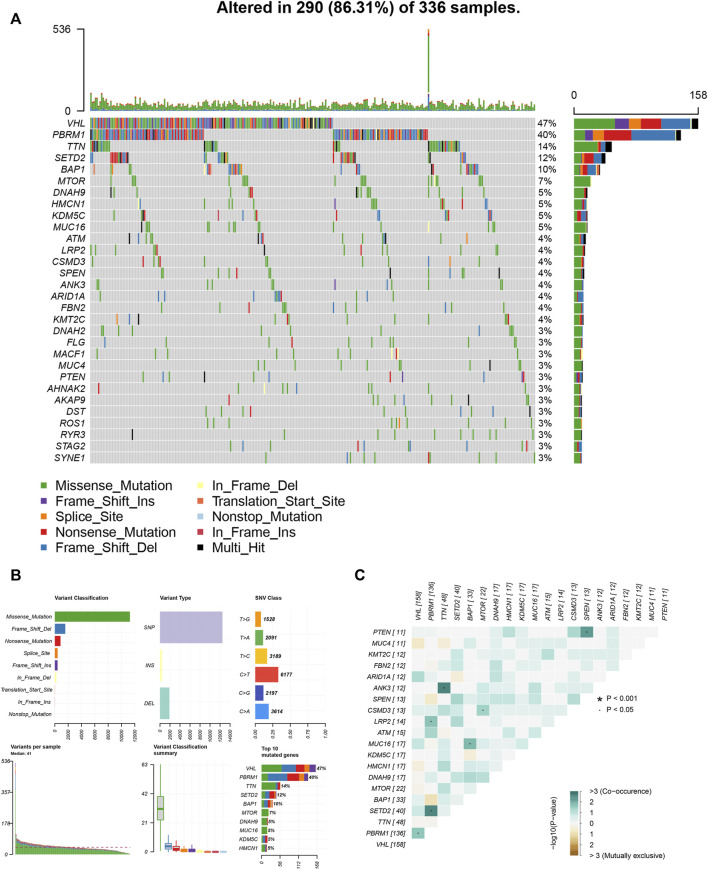
The association between ICI score and somatic variants. **(A)** The R package “maftools” analyzed the somatic gene variants in KIRC. **(B)** A detailed analysis of the variants. **(C)** The interaction among mutation genes.

We also assessed somatic variants of driver genes between the low and high ICI group and analyzed the mutation frequency using the R package “maftools.” The top 30 driver genes with higher mutation frequency were shown in Figure 5A. The results indicated that the mutation frequency of VHL, PBRM1, and TTN was high, which may provide some novel ideas concerning the association between ICI score and somatic mutation in immune checkpoint therapy (Figure 5B). The correlation between these mutation genes was also analyzed and the co-occurrence results were shown in Figure 5C.

### The Relationship Between the TMB, Immune Cell Infiltration Score, and Prognosis

**FIGURE 6:**
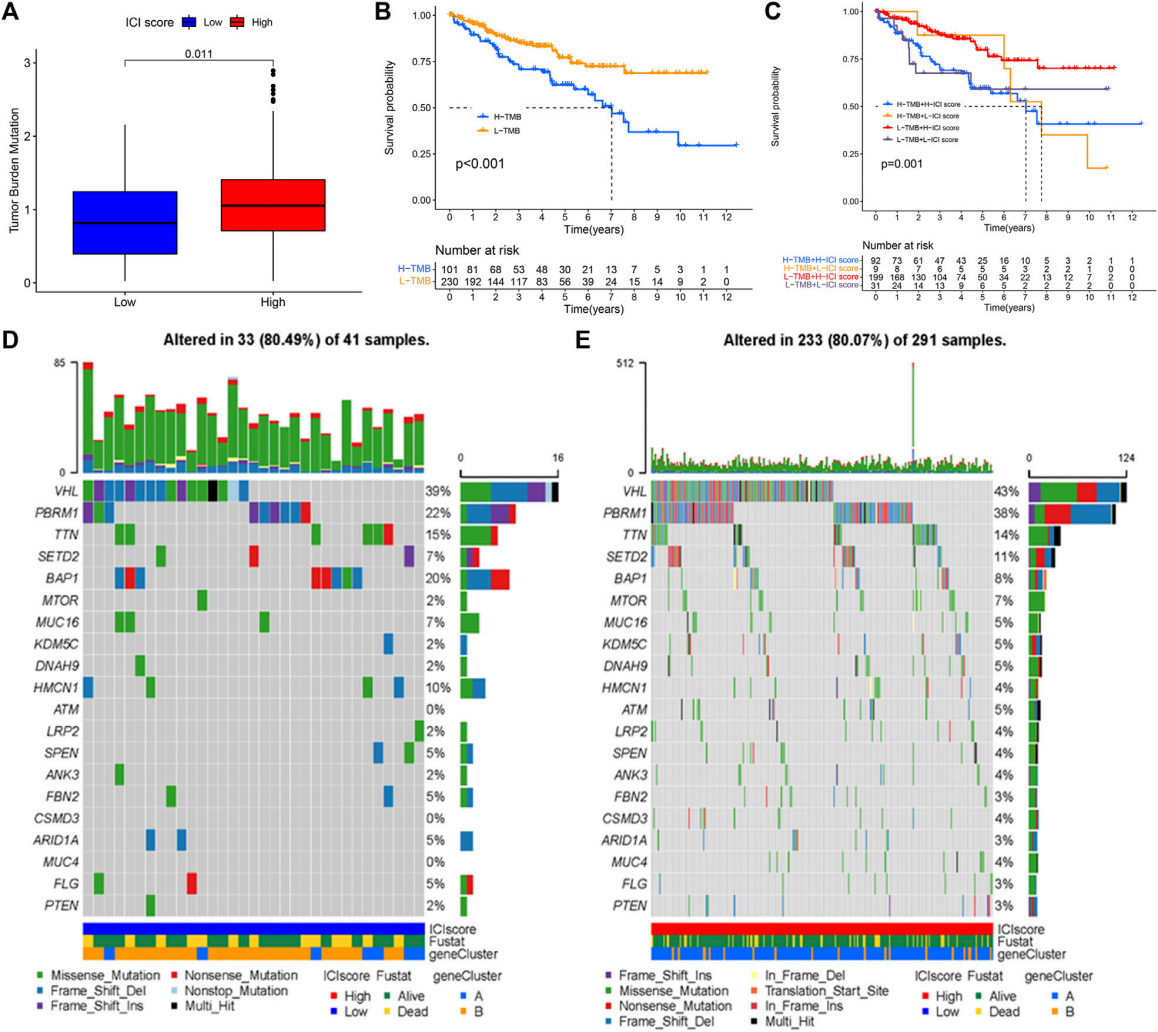
The association of TMB, ICI score, and survival status. **(A)** The high TMB was related to a high ICI score. **(B)** Kaplan-Meier curve for low and high TMB subgroups in KIRC cohort (log-rank test *p* < 0.001). **(C)** Kaplan-Meier curve analysis for different subgroups (log-rank test *p* = 0.001). **(D,E)** The top 20 driver genes in the low **(D)** and high **(E)** ICI score subgroups.

TMB has a relationship with TME and may be a predictor of immunotherapy response (Chan et al., 2019; Jardim et al., 2021; Strickler et al., 2021). TMB is all number of somatic mutations in a specific region of the tumor genome and varies by tumor type and patient (Stenzinger et al., 2019). The TMB values were listed in Supplementary Table S5. The high ICI scores were related to higher TMB (*p* = 0.011, Figure 6A). According to the median TMB value, the patients were divided into low and high TMB subtypes. Next, patients in the low TMB group exhibited better survival probability (log-rank test *p* < 0.001, Figure 6B). Subsequently, the patients were divided into four subgroups to assess the synergistic effect of ICI score and TMB in KIRC, including the high TMB + high ICI score subgroup, high TMB + low ICI score subgroup, low TMB + high ICI score subgroup, and low TMB + low ICI score subgroup. It can be observed that the prediction ICI score did not affect the TMB status, and the low TMB subgroups + high ICI score subgroups exhibited a better prognosis (log-rank test *p* = 0.001, Figure 6C). The somatic variants of driver genes in the low and high ICI score subgroups were determined using the R package “maftools.” The top 20 driver genes, especially VHL and PBRM1, with the highest mutation frequency in the low and high ICI score groups were shown in Figures 6D,E, respectively.

## Discussion

Immunotherapy can improve the anti-tumor immune response and has fewer off-target effects than chemotherapy and other drugs that directly kill cancer cells (Riley et al., 2019). Many cancer types have been treated using immunotherapy, such as melanoma, hepatocellular carcinoma, and breast cancer (Sugie, 2018; Yap et al., 2021). TME can regulate the hallmarks of cancer and provides good opportunities to use immunotherapy in the management of cancers (Lee and Cheah, 2019; Bejarano et al., 2021). At present, it is crucial to explore novel immune checkpoint therapeutic markers to identify the patient subgroups suitable for immunotherapy.

Based on TCGA data from KIRC tumor tissues, we identified four ICI subtypes. Based on the DEGs, three gene clusters were identified. We observed that the characteristics of TME and the proportion of immune infiltration cells were different among the ICI score subgroups, which revealed that ICI score plays a key role in the progression of tumors. A recent study also indicated ICI score was linked to patients’ prognosis and immune-related genes (Huang et al., 2021). Memory T cells and effector T cells are important immune defenses. T cells including CD4^+^ and CD8^+^ cells are involved in the immune responses and their aberrant regulatory behaviors contribute to autoimmune diseases (Brandt and Hedrich, 2018; Deng et al., 2019). The present study results showed that the infiltration levels of macrophages M1, plasma cells, activated dendritic cells, and activated mast cells were higher in ICI cluster A. ICI cluster B was marked by increased resting NK cells, monocytes, macrophages M2, and resting mast cells. Among the four ICI subgroups, the ICI cluster C group exhibited high infiltration level of CD8^+^ T cells, activated CD4 memory T cells, *γδ* T cells, activated NK cells, and immune scores and the expression levels of PD1 and PD-L1 were highest, which represent an immune-hot phenotype. ICI cluster D was marked with high macrophage M0 and had the shortest overall survival time. The infiltration levels of immune cells, such as macrophages, NK cells, dendritic cells, activated NK cells, and monocytes, are demonstrated to be indicators and biomarkers of clinical outcomes for multiple cancers (Song et al., 2019; Huang et al., 2022). ICI subgroup A and B, with a better prognosis, exhibited higher macrophages, activated dendritic cells, activated mast cells, NK cells, and monocytes.

Clinical immunotherapeutic approaches have led to a predictive response in a small number of cancer patients (Bu et al., 2020; Pan et al., 2020). Herein, among three gene subgroups, gene cluster C had the highest immune scores, exhibited higher *γδ* T cells, T cells follicular helper, and CD8^+^ T cells, and highest expression levels of PD1 and CTLA4, which replied that patients in this gene cluster may benefit from immunotherapy. The gene cluster A, with the worse prognosis, showed the lowest CD8^+^ T cells, low activated CD4 memory T cells, low *γδ* T cells, and low macrophages M1. The high immune infiltration of these immune cells by antigen-presenting cells may improve the patients’ prognosis in cluster A.

TME has individual heterogeneity, thus, further analyzing the ICI pattern and TME of individual tumors may provide novel ideas for promoting individualized immunotherapy. The ICI score was quantified to evaluate the degree of individual immune infiltration in KIRC. The enrichment results indicated that the B cell receptor signaling pathway, focal adhesion, T cell receptor signaling pathway, and TGF-β signaling pathway are enriched in the high ICI score subgroups, whereas, the tight junction is enriched in the low ICI score subgroup. We also observed that the low ICI score subgroup exhibited high expression of the most immune-related genes and poor prognosis. The results in bladder cancer indicated that the high ICI score group displayed higher immune-related genes and a good prognosis (Huang et al., 2021). These data suggest that different types of cancers may have an un-consistent expression of the immune checkpoint- and immune activity-related genes. In this study, the ICI score was observed to have a relationship with some clinical characteristics in KIRC patients. We observed that patients with grade 3/4, T3/T4, node metastasis, distant metastasis, and stage III/IV had low ICI scores, which revealed that low ICI scores may be associated with the development and poor prognosis of patients with KIRC. A recent study by Tang and Zhang (2021) provided constructive guides for our future studies, they conducted the hybrid nomogram including clinical features and the new pyroptosis-related lncRNAs prognostic signature and showed it was stable and accurate for treatment of clear cell renal cell carcinoma.

Somatic TMB, as a predictor biomarker in solid tumors, may result in neoantigen formation for immune system recognition (Sha et al., 2020; Cristescu et al., 2022). Furthermore, the TMB value was calculated and the relationship between ICI score and TMB was evaluated. The immune-related gene mutation and variants were analyzed and the results showed that VHL and PBRM1 were the two top mutated genes. The VHL and PBRM1 expression were correlated with tumor progression in several types of cancers and reflected the patients’ prognosis (Espana-Agusti et al., 2017; Högner et al., 2018). Combined with existing literature, VHL and PBRM1 may also be significantly related to the tumor progression of KIRC and may be used in immunotherapies for treating KIRC. Survival analysis indicated that the low TMB group had a better prognosis. The prognostic value of the TMB score was validated in KIRC cohorts. Several limitations exist in this study. For example, the present research is based on only one database, TCGA. Thus, another database International Cancer Genome Consortium (ICGC) will take into account in further investigation. In future studies, more validation with detailed driver genes combined with a nomogram is needed.

In conclusion, four ICI subtypes were identified based on ICI and the gene clusters were identified using the DEGs among ICI subtypes. ICI score and TMB were associated with the patients’ prognosis and might be effective prognostic biomarkers for evaluating immunotherapy response. The immune-related genes among ICI subtypes were explored. These results provided new insight for improving the immune response in patients treated with immunotherapies and providing clinicians with an array of evidence that they can optimally match to individual immune treatment based on their characteristics.

## Data Availability

The original contributions presented in the study are included in the article/Supplementary Material, further inquiries can be directed to the corresponding author.
