# Supplementary figures and images for: Identification of Immune-Related Subtypes and Characterization of Tumor Microenvironment Infiltration in Kidney Renal Clear Cell Carcinoma
Source: Front Genet. 2022 Jun 29;13:906113. doi: 10.3389/fgene.2022.906113 (PMC9277187; doi:10.3389/fgene.2022.906113)

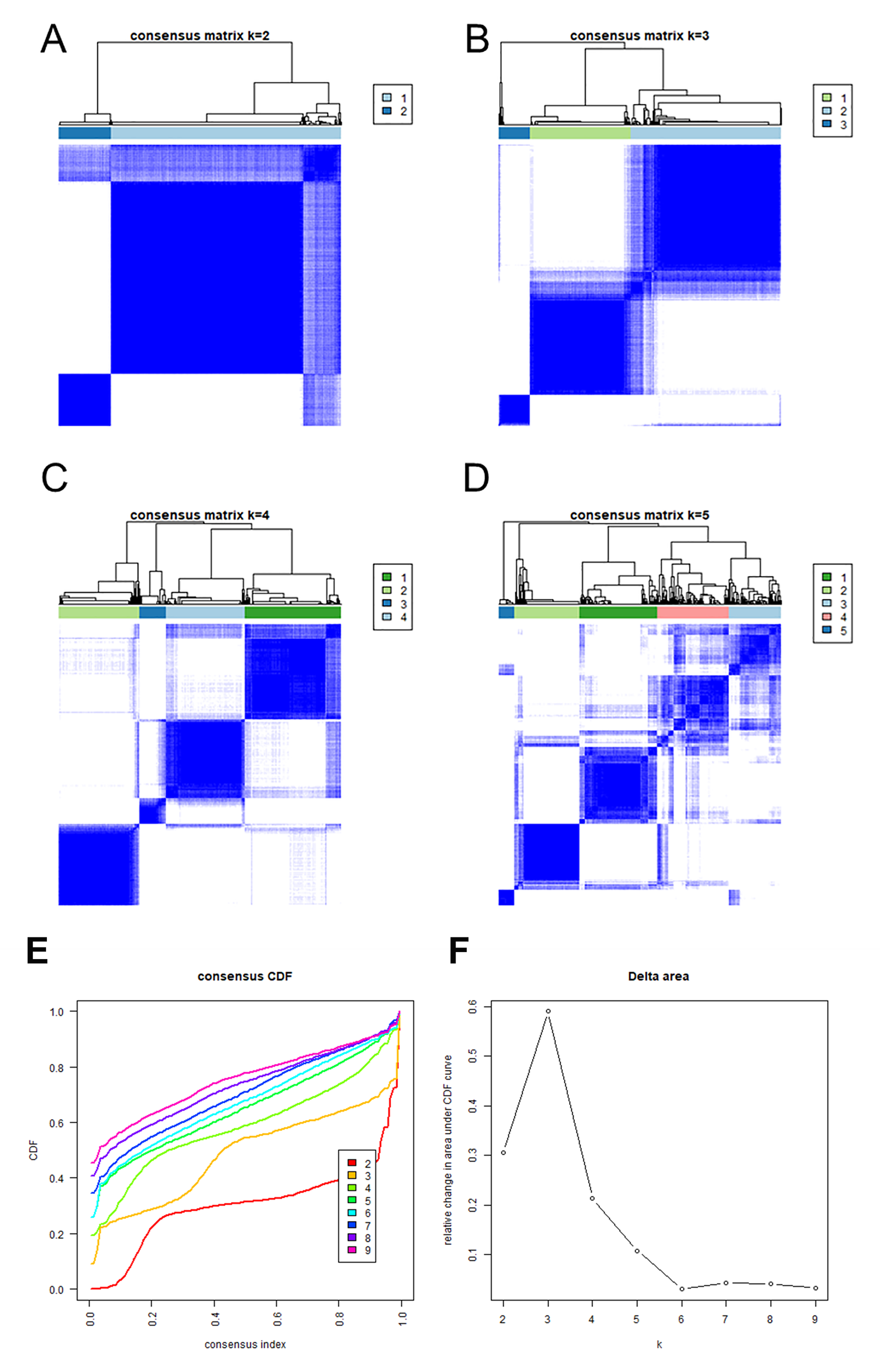

Supplement: Supplementary file 1 [file Image2.TIF]

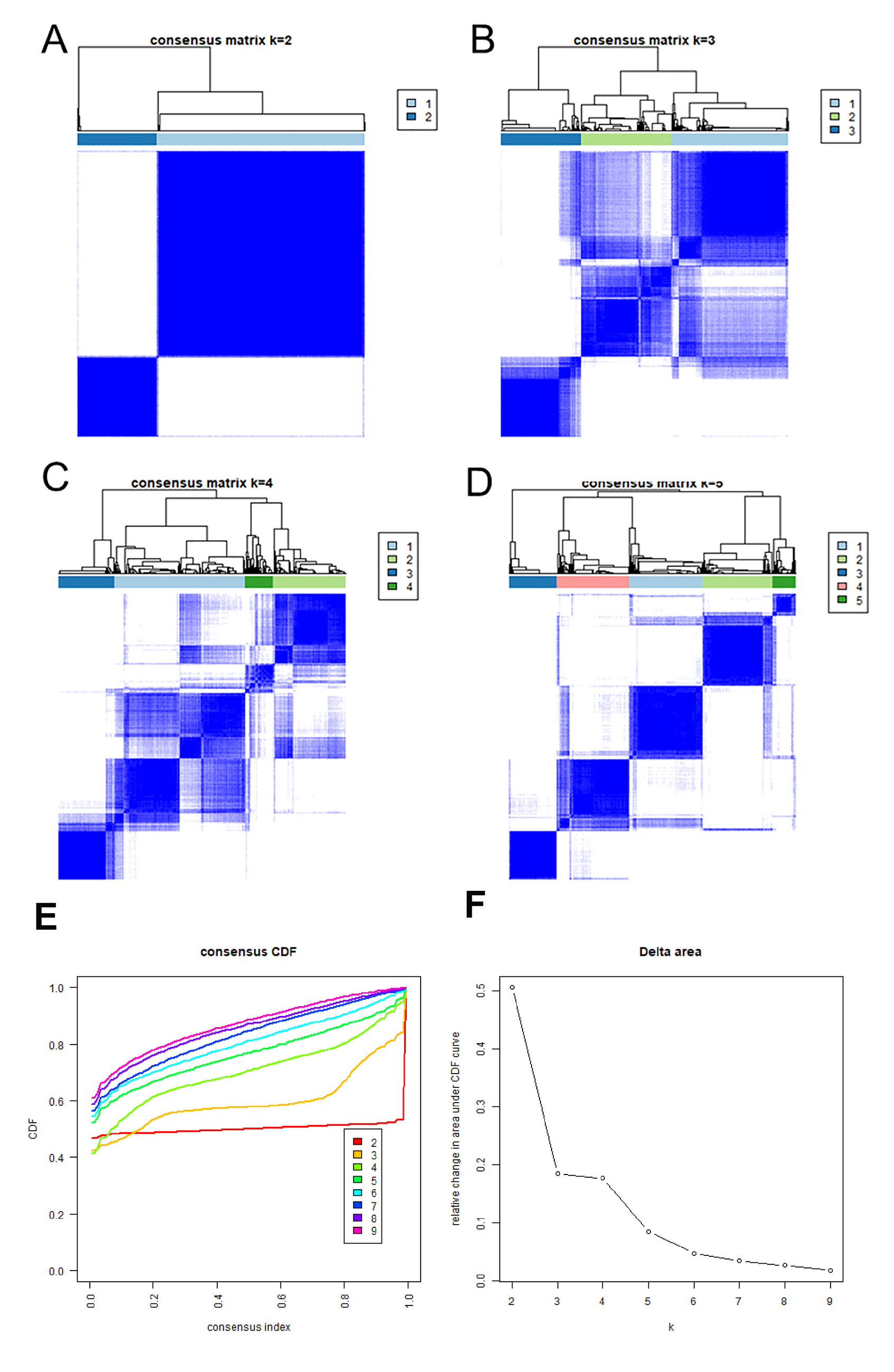

Supplement: Supplementary file 2 [file Image1.TIF]
